# Nicotine instigates podocyte injury via NLRP3 inflammasomes activation

**DOI:** 10.18632/aging.102611

**Published:** 2019-12-13

**Authors:** Gurinder Bir Singh, Naresh Kshirasagar, Sai Patibandla, Goverdhan Puchchakayala, Saisudha Koka, Krishna M. Boini

**Affiliations:** 1 Department of Pharmacological and Pharmaceutical Sciences, College of Pharmacy, University of Houston, Houston, TX 77204, USA

**Keywords:** inflammasome, podocyte, nicotine, caspase, WEHD

## Abstract

Background/Aims: Recent studies have shown that nicotine induces podocyte damage. However, it remains unknown how nicotine induces podocyte injury. The present study tested whether nicotine induces NLRP3 inflammasomes activation and thereby contributes to podocyte injury.

Results: Nicotine treatment significantly increased the colocalization of NLRP3 with Asc, caspase-1 activity, IL-β production, cell permeability in podocytes compared to control cells. Pretreatment with caspase-1 inhibitor, WEHD significantly abolished the nicotine-induced colocalization of NLRP3 with Asc, caspase-1 activity, IL-1β production and cell permeability in podocytes. Immunofluorescence analysis showed that nicotine treatment significantly decreased the podocin and nephrin expression compared to control cells. However, prior treatment with WEHD attenuated the nicotine-induced podocin and nephrin reduction. In addition, we found that nicotine treatment significantly increased the O_2_^.-^ production compared to control cells. However, prior treatment with WEHD did not alter the nicotine-induced O_2_^.-^ production. Furthermore, prior treatment with ROS scavenger, NAC significantly attenuated the nicotine-induced caspase-1 activity, IL-1β production, podocin and nephrin reduction in podocytes.

Conclusions: Nicotine-induced the NLRP3 inflammasome activation in podocytes and thereby results in podocyte injury.

Methods: Inflammasome formation and immunofluorescence expressions were quantified by confocal microscopy. Caspase-1 activity, IL-1β production and O_2_^.-^ production were measured by ELISA and ESR.

## INTRODUCTION

Cigarette smoking is a significant and well-known risk factor for many diseases of aging such as cardiovascular diseases, atherosclerosis and cancer. Smoking compromises both the life expectancy and the quality of life [1]. Previous studies have demonstrated that the development of kidney diseases could be associated with cigarette smoking [2, 3]. Particularly, in patients suffering from hypertension, diabetes induced polycystic kidney disease and kidney transplantation, cigarette smoking further worsens the progression of chronic kidney disease (CKD) [4–6].

Cigarette smoke contain thousands of compounds and among them nicotine is the key additive component [7]. Apart from its additive role, nicotine is shown to be involved in the pathogenesis of many diseases like aging, atherosclerosis, pulmonary fibrosis, obesity, hypertension and cancer [8, 9]. Recent studies have established the role of nicotine in renal diseases. Nicotine is associated with oxidative stress, enhanced mesangial proliferation, renal fibrosis and extracellular matrix deposition [10–12]. Nicotine was shown to promote glomerular injury in rodent models of acute nephritis and diabetic nephropathy [13]. In diabetic nephropathic mouse model, nicotine promotes extracellular matrix deposition resulting in renal injury [14]. Nicotine illicit its effects via nicotinic acetylcholine receptors (nAChRs) which are Ca^2+^ activated channels [14]. Nicotine also mediates its action by increasing oxidative stress in the kidney [6, 15] and renal proximal tubule cells in *in vitro* [16]. Previous reports indicate that α7nAChR is activated by nicotine in the proximal tubule. The active α7nAChR initiates the biosynthesis of profibrotic and proinflammatory cytokines [12]. However, the exact mechanism of how cigarette smoking accelerates the progression of CKD is still uncertain.

NLRP3 inflammasome is known to act as a sensor and is shown to be involved in inflammatory as well as non-inflammatory responses [17–21]. The pathogenic role of NLRP3 inflammasome has been established in several diseases like diabetes, silicosis, obesity, gout, acetaminophen-induced liver toxicity [22–29], unilateral ureteral obstruction [30, 31], acute ischemia/reperfusion-induced kidney injury [32], non-diabetic kidney disease [31] and obesity-induced glomerular injury [33]. The NLRP3 inflammasome is also activated by other triggers like bacterial toxins [34], monosodium urate crystals [23], cholesterol crystals [35], ATP, β-amyloid [36], visfatin [37], muramyl dipeptide [38] and other stimuli [22]. Recently, nicotine has been shown to be involved in development of inflammatory atherosclerotic plaques via NLRP3 inflammasome activation [39]. Hence, in the current study we tested whether nicotine activates NLRP3 inflammasomes in podocytes and contributes to podocyte damage.

## RESULTS

### Nicotine causes podocyte damage

**Figure 1:**
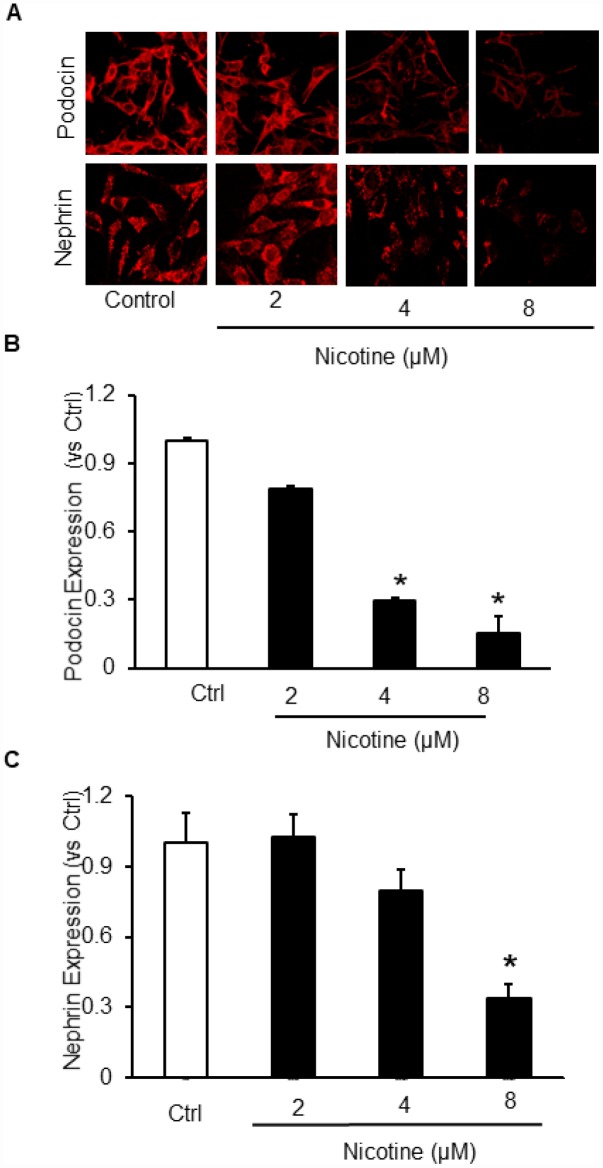
**Effect of Nicotine on podocyte injury.** Representative immunofluorescence images (**A**) and summarized quantification data shows Podocin (**B**) and Nephrin (**C**) expression in podocytes treated with different concentrations of Nicotine (2μM, 4μM, 8μM). Images were quantified using Image J software. N=5. * Significant difference from control.

To delineate the *in vitro* effects of nicotine on podocyte injury, cultured podocytes were treated with different concentrations of nicotine (2 μM, 4 μM and 8 μM) for overnight. Our results demonstrate that nicotine dose dependently decreased the expression of both podocin and nephrin which are markers of podocyte damage (Figure 1A, 1B). So, for further studies we selected 8 μM as an effective dose for remaining experiments.

### Activation of NLRP3 inflammasome by nicotine in cultured mouse podocytes

**Figure 2:**
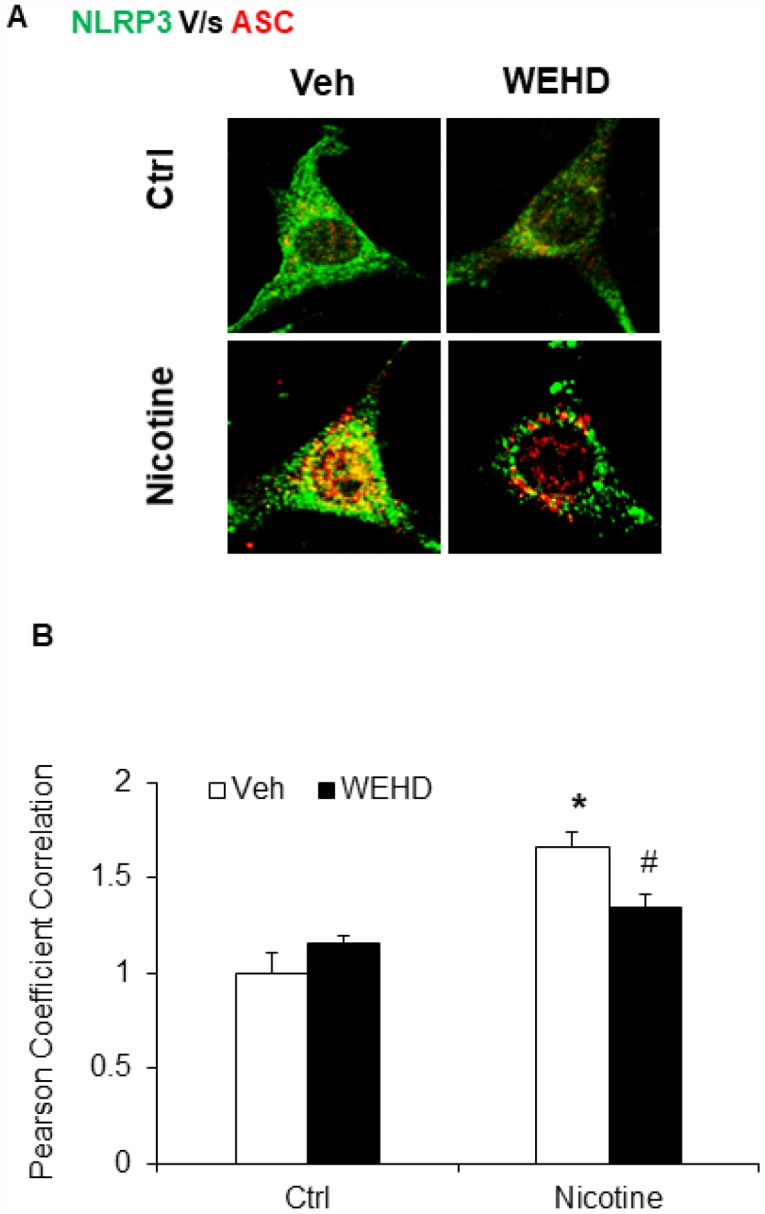
**Nicotine induced NLRP3 inflammasome formation in podocytes.** (**A**) Representative confocal fluorescence images show the colocalization of NLRP3 with ASC. (**B**) Summarized data shows the fold changes of pearson coefficient correlation (PCC) for the colo-calization of NLRP3 with ASC with or without stimulation of nicotine and/or caspase-1 inhibition by WEHD. N=5. Veh: Vehicle. *significant difference from control, ^#^ significant difference from nicotine treated group.

**Figure 3:**
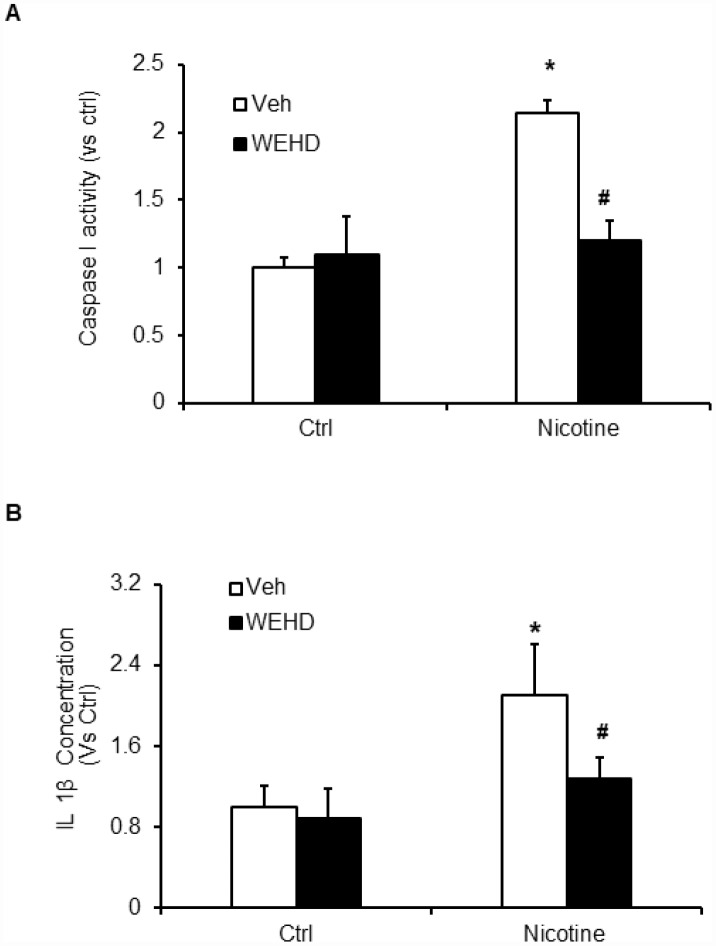
**Inflammasome activation by nicotine in podocytes.** Values are arithmetic means ± SEM (n=6 each group) of caspase-1 activity (**A**) and IL-β production (**B**) in podocytes with or without stimulation of nicotine and/or WEHD. *significant difference from control, ^#^significant difference from nicotine treated group.

We hypothesized that nicotine induces inflammasome activation leading to podocyte damage. As shown in Figure 2, nicotine induced co-localization of Nlrp3 (green) with ASC (red) as shown by increased yellow staining in podocytes. However, such nicotine-induced colocalization of Nlrp3 with Asc was blocked by WEHD, a caspase-1 inhibitor (Figure 2A). The summarized quantitative analysis of co-localization of Nlrp3 with Asc in podocytes is shown in Figure 2B. Furthermore, we have found that nicotine treatment enhanced the caspase-1 activity (Figure 3A) and increased production of IL-1β (Figure 3B) compared to control cells. Prior treatment of podocytes with caspase-1 inhibitor, WEHD attenuated nicotine-induced caspase-1 activity and IL-1β production (Figure 3A and 3B).

### Inhibition of caspase-1 protects the podocytes from nicotine-induced damage

**Figure 4:**
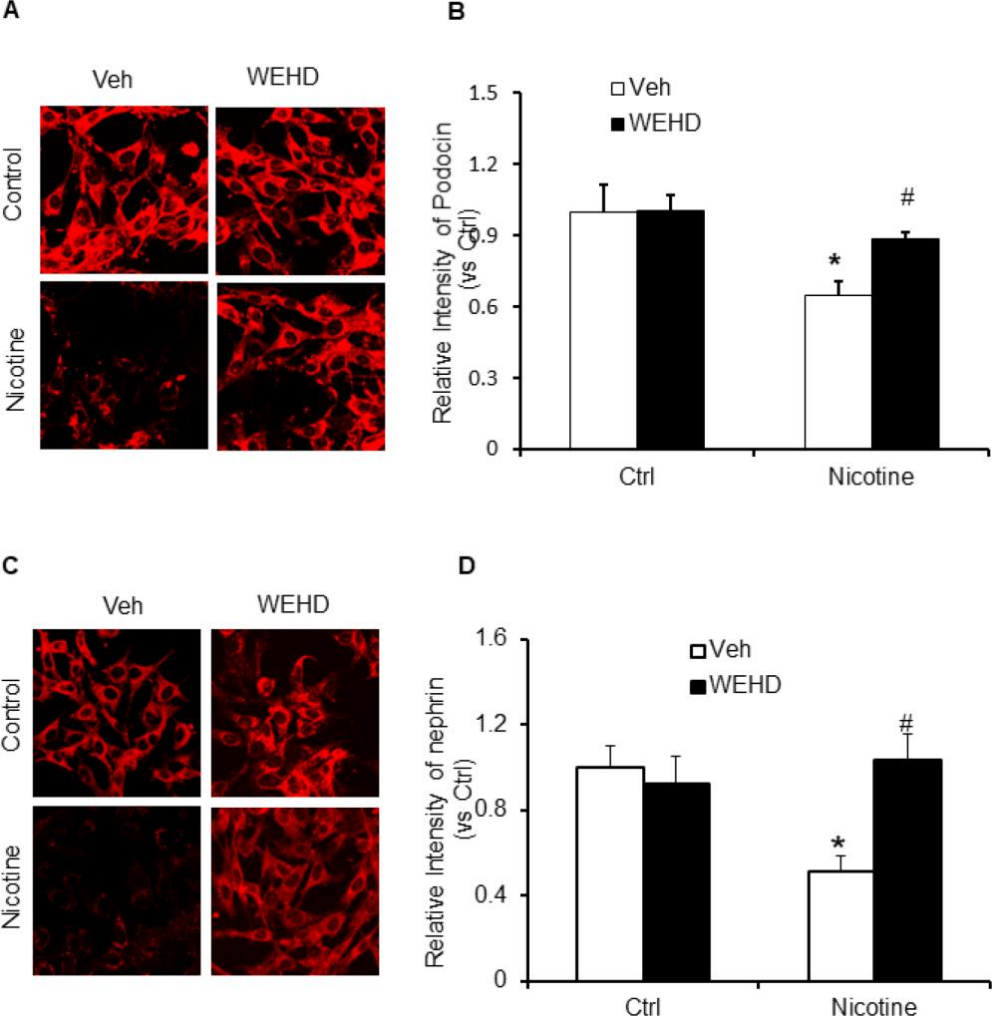
**Inhibition of inflammasome abolishes nicotine-induced podocyte injury.** Representative immunofluorescence images and summarized quantification data shows Podocin (**A**) and Nephrin (**B**) expression in podocytes treated with or without stimulation of nicotine and/or WEHD. Images were quantified using Image J software. N=5. *significant difference from control, #significant difference from nicotine treated group.

Podocyte-specific markers such as podocin and neprhin are down regulated during podocyte injury [33, 40]. Hence, the expression of podocin and desmin were monitored to assess the podocyte damage. Nicotine treatment resulted in an intense reduction of podocin and nephrin expression following immunofluorescence analysis demonstrating significant podocyte damage (Figure 4A–4D). Conversely, prior treatment with caspase-1 inhibitor, WEHD protected the podocytes from damage as shown by normalized protein expression of podocin and nephrin compared to control levels (Figure 4A–4D). These results confirm that nicotine is involved in podocyte dysfunction through the activation of NLRP3 inflammasomes in podocytes.

### Nicotine-induced podocyte monolayer permeability and dysfunction

**Figure 5:**
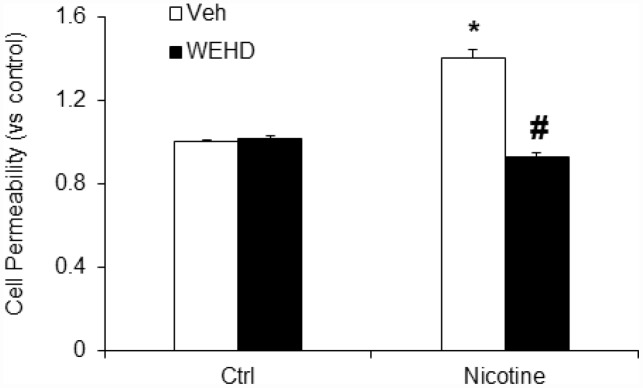
**Inhibition of inflammasome abolishes nicotine-induced cell permeability in podocytes.** Values are arithmetic means ± SEM (n=6 each group) of cell permeability in podocytes with or without stimulation of nicotine and/or WEHD. *significant difference from control, ^#^significant difference from nicotine treated group.

Next, we tested the role of nicotine in mediating podocyte dysfunction by examining its effect on the permeability of podocyte monolayers to FITC-dextran. As shown in Figure 5, nicotine significantly increased podocyte monolayer permeability as compared to untreated podocytes. Conversely, prior treatment with WEHD significantly attenuated the nicotine-induced increase in the podocyte monolayer permeability (Figure 5). This result suggests that nicotine is associated with podocyte monolayer disruption via NLRP3 inflammasomes.

### Nicotine-induced podocyte injury requires ROS

**Figure 6:**
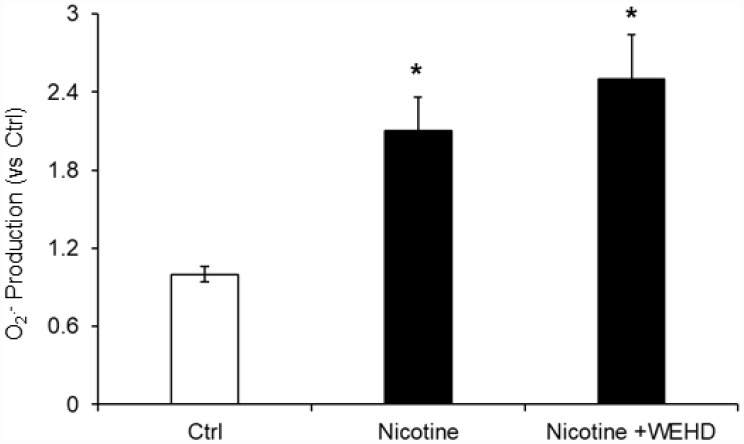
**O_2_^. –^ Production in podocytes with or without nicotine and/or WEHD treatment.** Values are arithmetic means ± SE (n=4 each group) of O_2_^. -^ production in podocytes with or without nicotine and/or WEHD treatment. Ctrl: Control, * Significant difference (*P*<0.05) compared to the control group.

The role of reactive oxygen species (ROS) in NLRP3 inflammasomes activation in different cell types is well established [20, 33]. Hence, we tested whether nicotine-induced O_2_^.-^ production in podocytes. We found that nicotine treatment significantly increased the O_2_^.-^ production compared to control cells. On the other hand, prior treatment with caspase-1 inhibitor, WEHD did not alter the nicotine-induced O_2_^.-^ production (Figure 6). This data suggests that nicotine-induced O_2_^.-^ production is upstream of NLRP3 inflammasomes activation.

**Figure 7:**
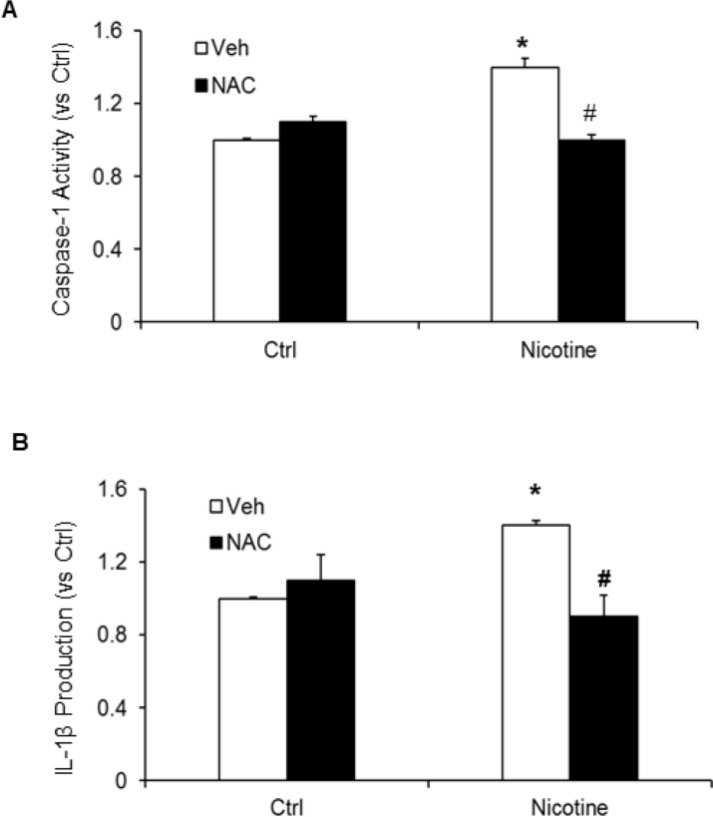
**Effect of NAC (N-acetyl-cysteine), ROS scavenger on nicotine-induced inflammasome activation.** Values are arithmetic means ± SEM (n=6 each group) of caspase-1 activity (**A**) and IL-β production (**B**) in podocytes with or without stimulation of nicotine and/or NAC. *significant difference from control, ^#^ significant difference from nicotine treated group.

**Figure 8:**
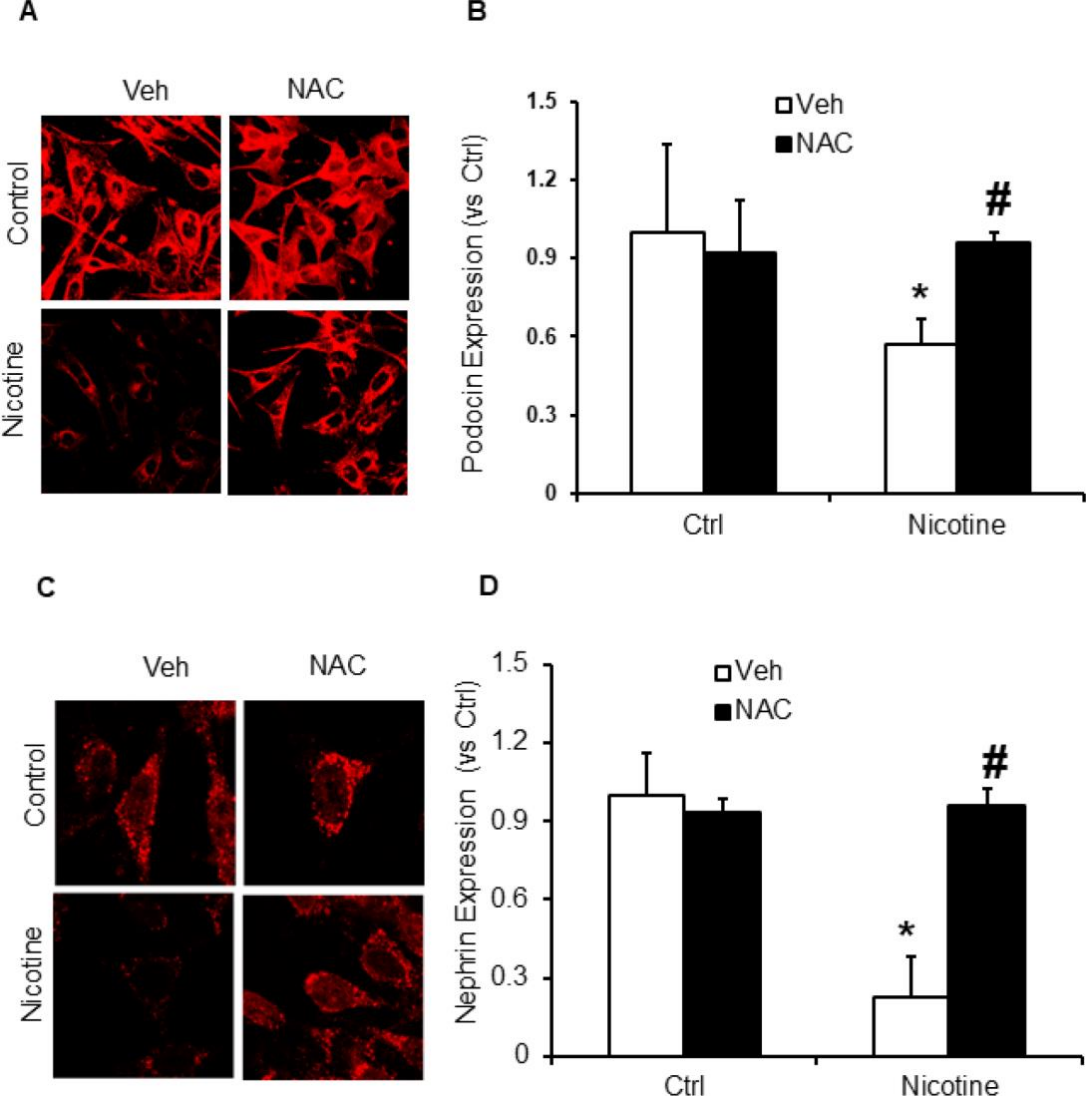
**ROS scavenger N-acetyl-cysteine (NAC) protected podocyte from nicotine-induced podocyte injury.** Representative immunofluorescence images and summarized quantification data (n=6 each group) shows podocin (**A**) and nephrin (**B**) expression in podocytes treated with or without stimulation of nicotine and/or NAC. Images were quantified using Image J software. *significant difference from control, ^#^significant difference from nicotine treated group.

Subsequently, we examined whether, N-acetyl- cysteine (NAC), a scavenger of ROS attenuates nicotine induced inflammasome activation and podocyte damage. As shown in Figure 7, the activity of caspase-1 and production of IL-1β was significantly increased in podocytes upon nicotine treatment. However, prior treatment with ROS scavenger NAC attenuated the activity of caspase-1 activity and production of IL-1β induced by nicotine (Figure 7). Furthermore, we also found that NAC rescued podocyte injury as evident from restored podocin and nephrin expression in podocytes treated with nicotine along with NAC (Figure 8).

## DISCUSSION

The major goal of our current study was to identify whether activation of inflammasomes contributes to nicotine induced podocyte damage and consequent glomerular sclerosis. Our results demonstrate that nicotine induced aggregation of NLRP3 inflammasome components, caspase-1 activation and IL-1β production leading to injury of the cultured podocytes. However, such activation of inflammasome and podocyte injury by nicotine was attenuated in WEHD treated cells. In addition, we also confirmed the role of ROS in nicotine associated podocyte damage via activation of NLRP3 inflammasomes. Our results demonstrate the critical role of nicotine in turning on inflammatory response via activating NLRP3 inflammasomes leading to renal inflammation and renal dysfunction during smoking and may help in understanding the pathophysiology of nicotine associated chronic kidney diseases.

Cigarette smoking is known to accelerate the rate of development of chronic kidney diseases, polycystic kidney disease, diabetes mellitus [2, 41], hypertension [4, 5], IgA nephropathy, lupus nephritis [42], and post kidney transplantation [3, 6]. Likewise, investigational studies revealed that the exposure to environmental tobacco smoke worsens renal injury in diabetic nephropathy and aging mouse models [12]. In another study, it was shown that nicotine causes the podocyte damage through JNK, ERK1/2, and p38 pathways that are regulated ROS generation [43]. However, despite the existing experimental and clinical data, the exact mechanisms responsible for the deleterious effects of nicotine have not been identified. Nevertheless, NLRP3 inflammasome activation has been recently associated with various pathological conditions such as gout, diabetes, chronic kidney diseases including podocyte damage and glomerular injury [17, 23–25, 27–30, 32, 33] and to a number of other diseases like acute lung injury, silicosis, Alzheimer’s disease, liver toxicity, and cystic fibrosis [22–25, 27, 28]. Yet, it is not known whether nicotine activates NLRP3 inflammasome and contributes to podocyte damage. In the present study, we have shown that colocalization of NLRP3 with ASC, caspase-1 activity and IL-1β production was increased by nicotine stimulation suggesting the formation and activation of NLRP3 inflammasomes. This nicotine-induced inflammasome formation and activation was abolished in podocytes with prior treatment with caspase-1 inhibitor, WEHD (Figure 2 and 3). Thus, our results indicate that nicotine enhanced activation of Nlpr3 inflammasome in podocytes, which may contribute to the development of podocyte damage. These results are consistent with earlier reports that nicotine treatment increased the expression of NLRP3, ASC, caspase-1 activation and IL-1β production in human aortic endothelial cells (HAECs) and silencing NLRP3 and ASC in endothelial cells by RNA interference (siRNA) significantly attenuated the nicotine-induced activation of NLRP3, ASC, caspase-1, IL-1β and IL-18 production [39]. To our knowledge, the current study provides the first experimental evidence indicating that nicotine induced NLRP3 inflammasome formation and activation in podocytes. Our results clearly indicate that nicotine induced activation of NLRP3 inflammasomes in podocytes contributes to the development of kidney diseases.

During physiological and pathological processes, podocytes play a critical role in the prevention of glomerular protein leakage through the formation of slit diaphragm [43]. Smoking worsens the kidney diseases and clinical studies have demonstrated that smoking enhances proteinuria [11, 43]. Hence, it could be possible that the tobacco smoke contents directly affect the podocytes in the kidney [43]. In this context, we demonstrated that nicotine reduced the nephrin expression in podocytes, signifying that it triggered podocyte injury [43]. However, it remains unknown whether inhibition of inflammasome by WEHD protects against the nicotine-induced podocyte injury. In the present study, we found that nicotine treatment significantly reduced podocin and nephrin expression in podocytes, which was abolished by prior treatment with WEHD, a caspase-1 inhibitor suggesting the critical role of NLRP3 inflammasomes in nicotine associated podocyte injury (Figure 4). Functional significance of nicotine associated NLRP3 activation was further explored by studying nicotine-induced enhancement of podocyte monolayer permeability. Increased vascular permeability resulting in increased glomerular permeability is known to contribute to the development and advancement of glomerular injury [44, 45]. The present study demonstrates that nicotine increased the permeability of podocyte monolayer and this nicotine-induced increase in podocyte permeability was significantly attenuated by treatment with a caspase-1 inhibitor, WEHD, suggesting the role of NLRP3 inflammasomes in nicotine associated increase in podocyte permeability (Figure 5).

Next, we confirmed the mechanisms underlying nicotine induced NLRP3 inflammasome activation in podocytes. Inflammasome activation is known to sense changes in cellular oxidative stress and activation of the NLRP3 inflammasome by increased ROS is the most widely accepted and reasonably known mechanism [20, 46, 47]. Consistent with these reports, our study demonstrated that treatment with ROS scavenger, N-acetyl-cysteine (NAC) attenuated nicotine-induced caspase-1 activity as well as IL-1β production in podocytes. NAC treatment protected podocytes from ROS induced injury as demonstrated by the preserved expression of podocin and nephrin in nicotine treated cells, suggesting the role of ROS in nicotine induced NLRP3 inflammasome activation (Figures 7 and 8). However, further studies are required to reveal which nicotinic receptors are mediating the inflammasome activation in podocytes. In this regard, there were reports that nicotine mediates its effects through the activation of different nicotinic acetylcholine receptors (nAChRs) in different cell types including α4, α5, α7, β2, β3 and β4 which are expressed in human mesangial cells [14, 43]. α3, α5, and β1 are expressed in renal proximal tubular epithelial cells (HK-2), α5, α6, α7 and β3 are expressed in podocytes [43, 48]. Recently Lu et al reported that α7 nAChR activation contributed to the NLRP3 inflammasome inhibition in peritoneal mouse macrophages and dendritic cells [49]. On-going studies in our laboratory will further clarify the role of these nicotine subunits on nicotine-induced activation of inflammasome activation and podocyte damage. In conclusion, the current study confirmed for the first time that nicotine-induced NLRP3 inflammasome activation in podocytes and thereby results in podocyte injury. Hence, targeting Nlrp3 inflammasome might be a promising therapeutic approach to inhibit inflammasome activation and protect podocytes against nicotine-induced injury.

## MATERIALS AND METHODS

### Cell culture

A conditionally immortalized mouse podocyte cell line was cultured undifferentiated with 10 U/ml recombinant mouse interferon-γ at 33°C on collagen I-coated flasks in serum containing RPMI 1640 medium containing 10% fetal bovine serum, 100 U/ml penicillin and 100 mg/ml streptomycin. After differentiation at 37°C for 10–14 days without interferon–γ, podocytes were used for the following experiments. Podocytes were pretreated with WEHD or N-acetyl cysteine (NAC) (10μM) for 30 min prior to nicotine treatment (8μM) for overnight.

### Immunofluorescence staining of cells

Cells were fixed in 4% PFA, and then blocked with 1% BSA for 30 min. After incubated with primary antibody (podocin, nephrin, NLRP3 and ASC) 1:200 dilution in 0.1% BSA overnight, the cells were stained with secondary antibodies. After washing, the cells were mounted with DAPI-containing mounting solution. Then the slides were observed under a fluorescent microscope and images were taken (Nikon, Japan). For, confocal analysis, the slides were visualized through sequentially scanning on Leica laser scanning confocal microscope (Leica SP-8, Germany). Colocalization was analyzed by Image J, and the co-localization coefficient was represented by Pearson's correlation coefficient [40].

### Caspase-1 activity and IL-1β production assay

After nicotine treatment, cells were harvested and homogenized to extract proteins for caspase-1 activity assay by using a commercially available kit (Biovision). These data were expressed as the fold change compared with control cells. In addition, the cell supernatant was also collected to measure the IL-1β production by a mouse IL-1β ELISA kit (Bender Medsystems, Burlingame, CA) according to the protocol described by the manufacturer [21, 50].

### Electronic spin resonance (ESR) analysis of O_2_^.-^ production

For detection of O2^.-^ production, proteins from podocytes were extracted using sucrose buffer and resuspended with modified Kreb’s–Hepes buffer containing deferoximine (100 mM, Sigma) and diethyldithiocarbamate (5 mM, Sigma). The podocytes (20 μg protein) were incubated for 10 min at 37 °C in the presence or absence of SOD (200 U/ml), and then supplied with 1mM O2 ^.-^ specific spin trap 1-hydroxy-3-methoxycarbonyl-2,2,5,5-tetramethylpyrrolidine (CMH, Noxygen, Elzach, Germany). The mixture was loaded in glass capillaries and immediately analyzed for O2 ^.-^ production kinetically for 7 min in a Miniscope MS5000 electromagnetic spin resonance [33, 37, 45] spectrometer (Rotunda Scientific Technologies, Stow, Ohio). The results were expressed as the fold changes of control.

### Cell permeability assay

The permeability of podocyte layer in culture was measured according to the methods as described previously [19]. In brief, podocyte were seeded in the upper chambers of 0.4 μm polycarbonate transwell filters of a 12-well filtration microplate (Whatman Inc.). After reaching confluence, the culture medium was replaced with fresh serum free RPMI 1640 media in presence of nicotine (8μM) with or without WEHD and incubated overnight. Following day fresh phenol red-free RPMI 1640 with 70 kDa FITC-dextran (2.5 μmol/l) in the upper chambers was added and incubated for 2.5 hrs. The filtration microplate was removed and the medium from the lower compartment was collected, and then fluorescence was measured in a spectrofluorimeter at 494 nm excitation and 521 nm emissions. The relative permeable fluorescence intensity was used to represent the cell permeability [45].

### Statistical analysis

Data are provided as arithmetic means ± SEM; *n* represents the number of independent experiments. All data were tested for significance using ANOVA or paired and unpaired Student’s t-test as applicable. Only results with p<0.05 were considered statistically significant.
